# Supplementary figures and images for: Erg251 has complex and pleiotropic effects on sterol composition, azole susceptibility, filamentation, and stress response phenotypes
Source: PLoS Pathog. 2024 Jul 30;20(7):e1012389. doi: 10.1371/journal.ppat.1012389 (PMC11315318; doi:10.1371/journal.ppat.1012389)

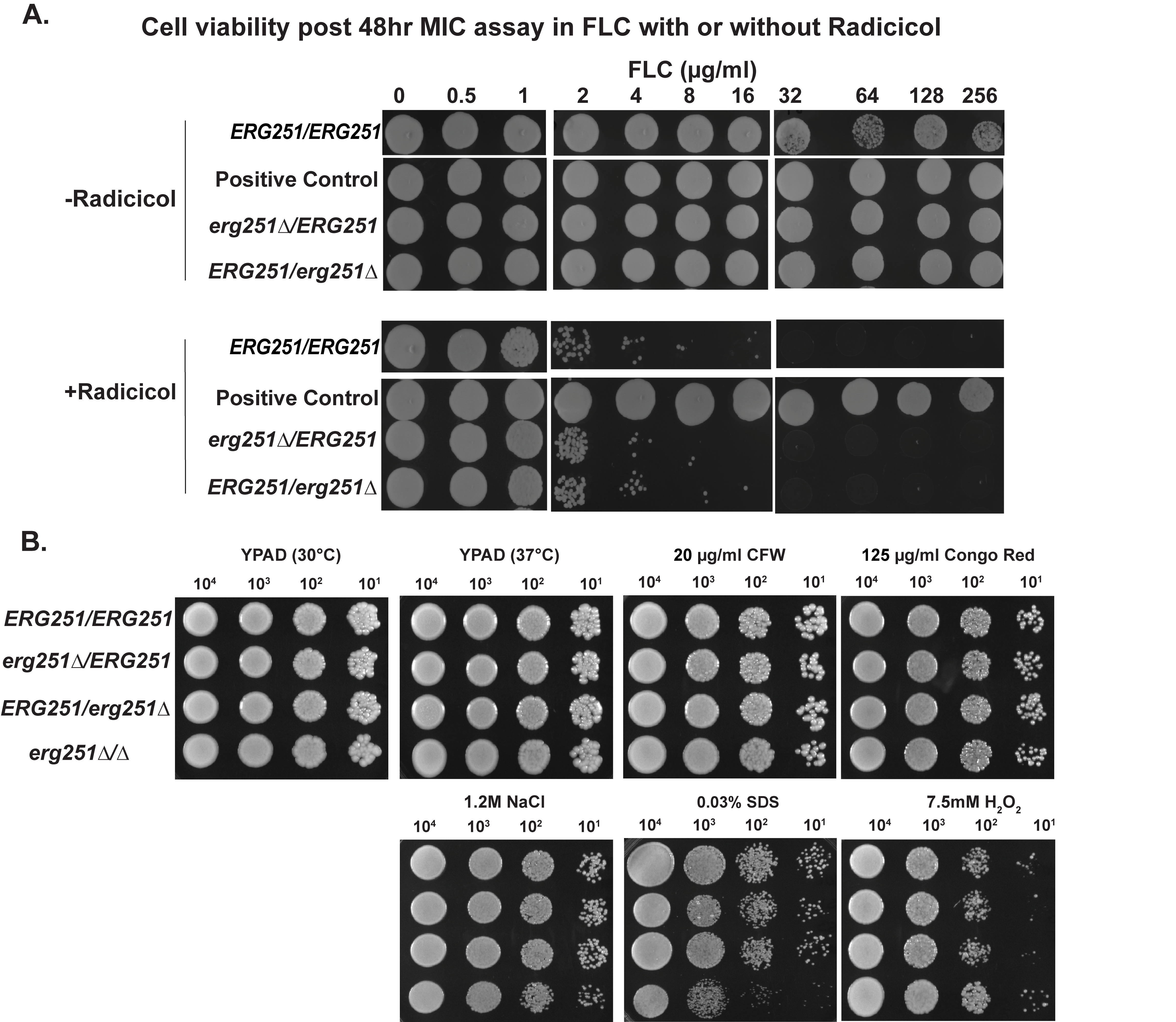

Supplement: S2 Fig — A. Cells from the MIC assay at 48 hr in Fig 1D, with or without radicicol, were plated for viability on YPAD agar plates and imaged after 24 hr incubation. Wild-type SC5314 (ERG251/ERG251), and both heterozygous deletion mutants of ERG251 were tested with a FLC resistant clinical isolate (C17/12-99, S1 Table) as a positive control. B. Spot plates growth of ERG251/ERG251, erg251Δ/ERG251, ERG251/erg251Δ, and erg251Δ/Δ on YPAD (30°C), YPAD (37°C), 20 μg/ml calcofluor white (CFW), 125 μg/ml Congo Red, 1.2M NaCl, 0.03% SDS and 7.5mM H2O2 agar plates. A & B. At least three biological replicates were performed. (TIF) [file ppat.1012389.s002.tif]

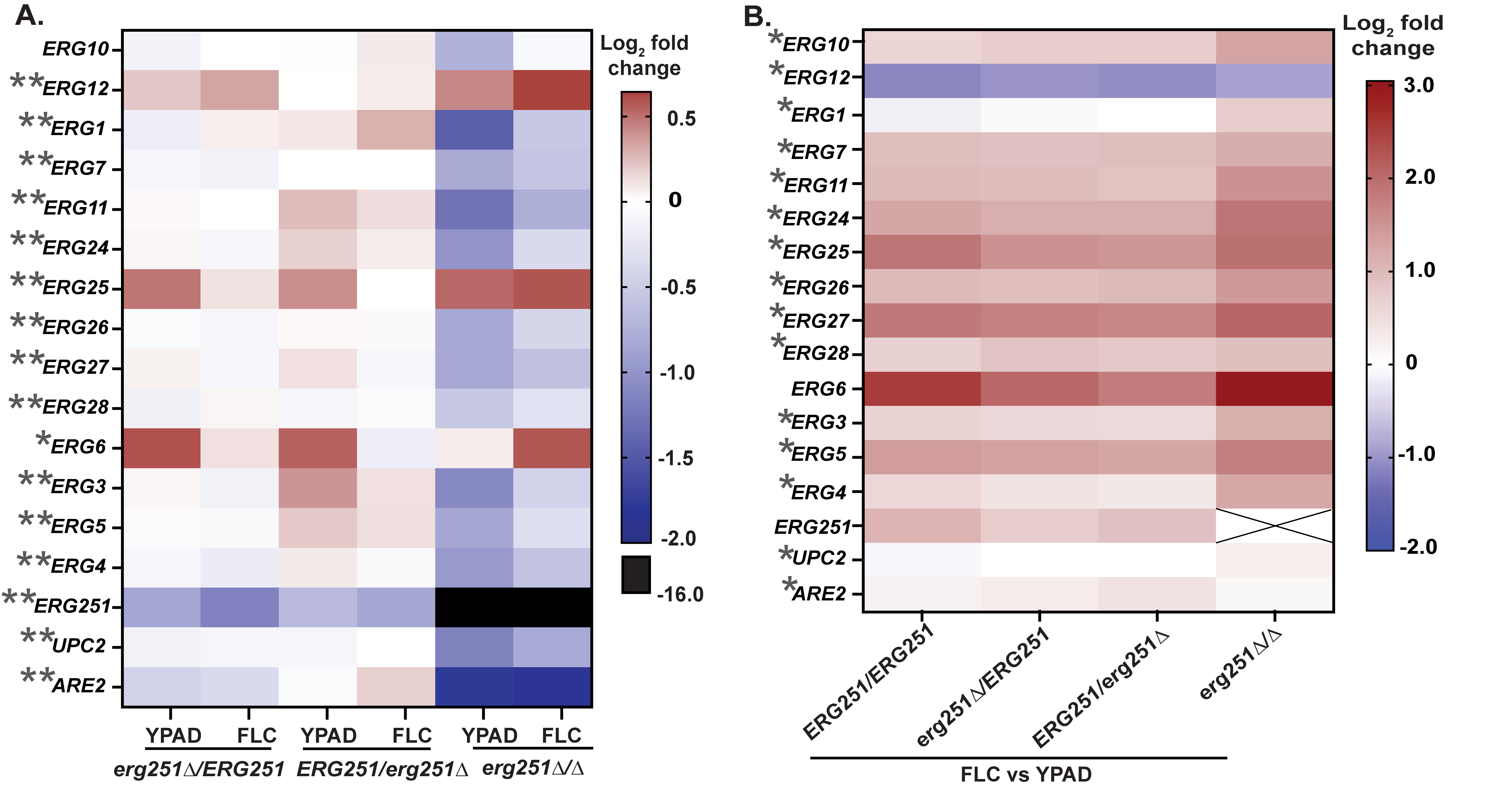

Supplement: S4 Fig — A. The relative gene expression levels (log2-fold change) for all ERG genes in the heterozygous and homozygous mutants erg251Δ/ERG251, ERG251/erg251Δ, and erg251Δ/Δ grown in YPAD or YPAD+1μg/ml FLC conditions, compared to the wildtype ERG251/ERG251 in the same condition. Two asterisks indicate the expression change is significant (adjusted p-value < 0.05) in erg251Δ/Δ relative to ERG251/ERG251 in both YPAD (S2 Table) and YPAD+1μg/ml FLC (S11 Table) conditions. One asterisk indicates ERG6 expression level change is significant (adjusted p-value < 0.05) in erg251Δ/Δ relative to ERG251/ERG251 only in YPAD+1μg/ml FLC condition (S11 Table). B. The relative expression level (log2 fold change) of ERG genes in the wildtype ERG251/ERG251, and mutants erg251Δ/ERG251, ERG251/erg251Δ, and erg251Δ/Δ grown in YPAD+1μg/ml FLC compared to YPAD condition. One asterisk indicates the expression change is significant (adjusted p-value < 0.05) in the erg251Δ/Δ in YPAD relative to erg251Δ/Δ in YPAD+1μg/ml fluconazole conditions (S15 Table). (TIF) [file ppat.1012389.s004.tif]

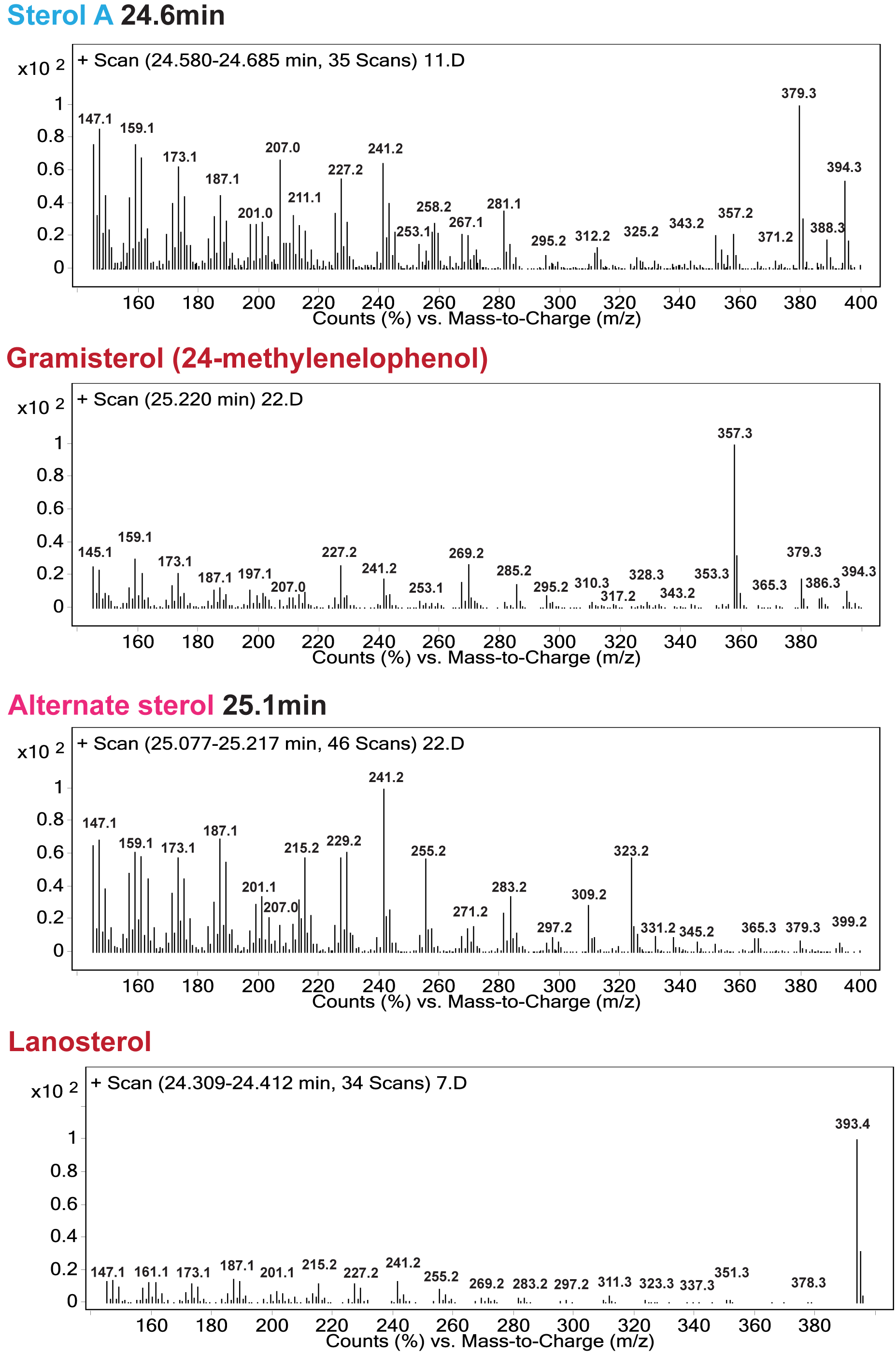

Supplement: S5 Fig — Mass spectra of two unidentified sterols: Sterol A (24.6 min) and the Alternate sterol (25.1 min) from Fig 6B, and two related standards Gramisterol (Smolecule, catalog # S626191) and Lanosterol (Smolecule, catalog #S532452). (TIF) [file ppat.1012389.s005.tif]

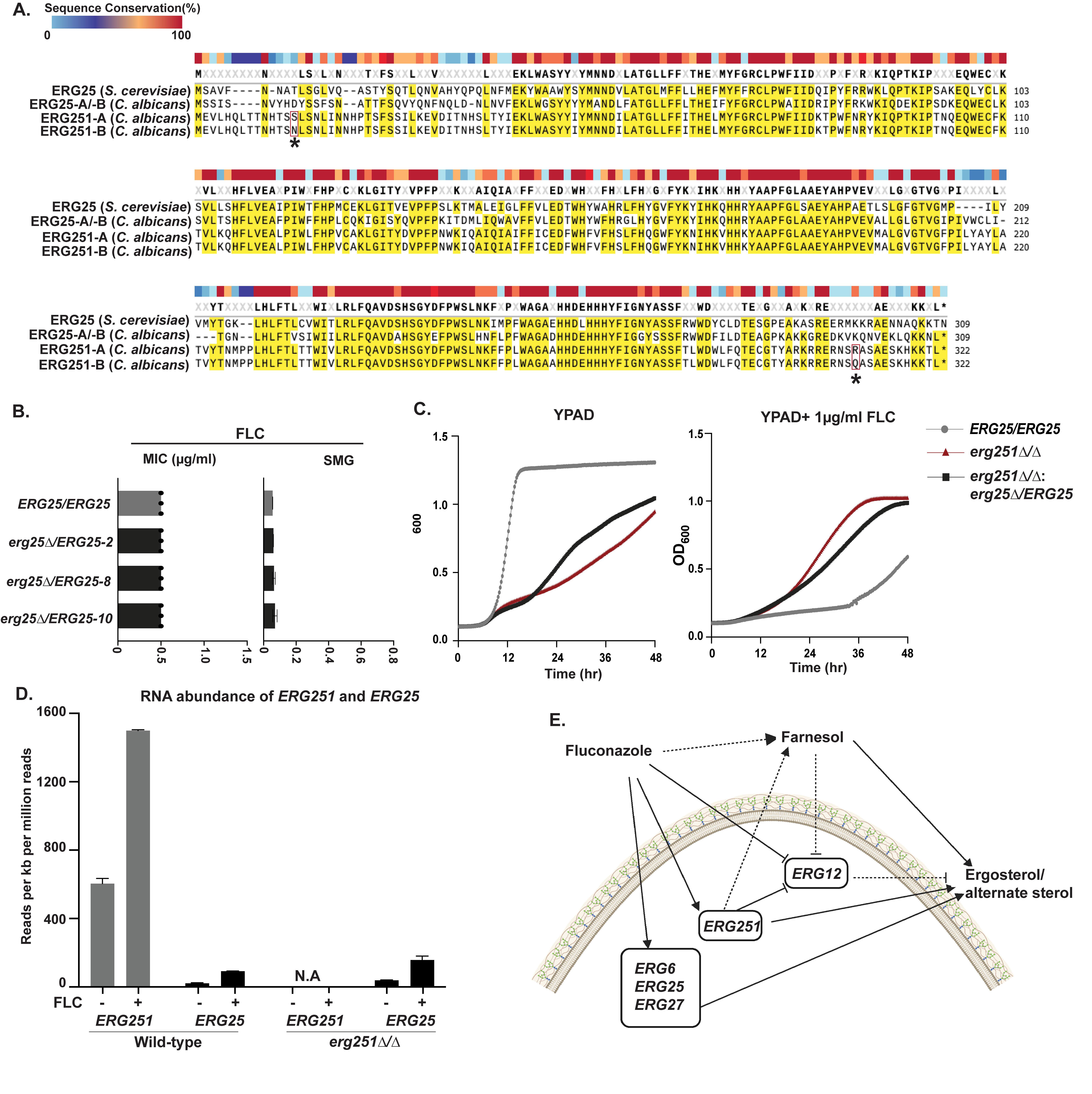

Supplement: S6 Fig — (A) Multiple sequence alignment for ERG251-A, ERG251-B, and ERG25-A/-B (no SNPs between A and B) from C. albicans and ERG25 from S. cerevisiae, with yellow highlighting similarity among all four proteins. Colored blocks on the top indicate the sequence conservation. Asterisks (*) and red boxes indicate the locus of non-synonymous variation between ERG251-A and ERG251-B in C. albicans. B. FLC susceptibility determined by liquid microbroth dilution at 24hr MIC (left, μg/ml) and 48hr SMG (right, tolerance) in FLC for three ERG25 heterozygous deletion mutants (ERG25/erg25Δ-2, -8 and -10) in the SC5314 background with SC5314 (ERG25/ERG25) as the control. MIC: each dot represents a single replicate and bar represents the average of three biological replicates of a single strain; SMG values are mean ± SEM calculated from three biological replicates of a single strain. C. 48hr growth curve analysis of erg25 heterozygous deletion strain in erg251Δ/Δ background (erg251Δ/Δ: ERG25/erg25Δ) in YPAD (left) and YPAD+1μg/ml FLC (right) with SC5314 (ERG25/ERG25) and erg251Δ/Δ as the controls. The initial cell densities were OD600 of 0.001. MIC and SMG are not measurable for erg251Δ/Δ or erg251Δ/Δ: ERG25/erg25Δ given growth defects in YPAD. B&C: Minimum of three biological replicates were performed. D. RNA abundance of ERG251 and ERG25 in SC5314 (wild-type), and in erg251Δ/Δ. RNA reads were normalised to transcript length and total RNA reads. Values are mean ± SEM calculated from three biological replicates. E. Predicted model for how FLC and farnesol impact the expression of ERG genes. In the wild-type, low concentrations of FLC promotes the expression of most ERG genes, including ERG6, ERG251, ERG25, ERG11 and ERG27, leading to the upregulation of ergosterol or/and alternate sterol biosynthesis. However, both low concentrations of FLC and Erg251 pose a negative regulation on Erg12, which may be achieved via farnesol which we predict inhibits ERG12 [90]. Dashed lines indicate predict [file ppat.1012389.s006.tif]
