# Supplementary material for: Erg251 has complex and pleiotropic effects on sterol composition, azole susceptibility, filamentation, and stress response phenotypes
Source: PLoS Pathog. 2024 Jul 30;20(7):e1012389. doi: 10.1371/journal.ppat.1012389 (PMC11315318; doi:10.1371/journal.ppat.1012389)
Supplement: Attachment — Submitted filename: 2024_05_24_ERG251_ResponseLetternew.pdf [file ppat.1012389.s024.pdf]

Dear Dr. Selmecki,

Thank you very much for submitting your manuscript "Erg251 has complex and pleiotropic effects on azole susceptibility, filamentation, and stress response phenotypes" for consideration at PLOS Pathogens. As with all papers reviewed by the journal, your manuscript was reviewed by members of the editorial board and by several independent reviewers. In light of the reviews (below this email), we would like to invite the resubmission of a significantly-revised version that takes into account the reviewers' comments.

While the reviewers were generally positive about this work and its significance, they have identified a number of issues that need to be addressed. These will require additional experimentation and revision of this manuscript.

The companion submission to your manuscript has also been evaluated on its merits, and we have reached a different decision based on the reviewers' recommendation. Therefore, we cannot guarantee to publish the two submissions side-by-side. We cannot make any decision about publication until we have seen the revised manuscript and your response to the reviewers' comments. Your revised manuscript is also likely to be sent to reviewers for further evaluation.

When you are ready to resubmit, please upload the following:

[1] A letter containing a detailed list of your responses to the review comments and a description of the changes you have made in the manuscript. Please note while forming your response, if your article is accepted, you may have the opportunity to make the peer review history publicly available. The record will include editor decision letters (with reviews) and your responses to reviewer comments. If eligible, we will contact you to opt in or out.

[2] Two versions of the revised manuscript: one with either highlights or tracked changes denoting where the text has been changed; the other a clean version (uploaded as the manuscript file).

Important additional instructions are given below your reviewer comments.

Please prepare and submit your revised manuscript within 60 days. If you anticipate any delay, please let us know the expected resubmission date by replying to this email. Please note that revised manuscripts received after the 60-day due date may require evaluation and peer review similar to newly submitted manuscripts.

Thank you again for your submission. We hope that our editorial process has been constructive so far, and we welcome your feedback at any time. Please don't hesitate to contact us if you have any questions or comments.

Sincerely,

Chaoyang Xue, Ph.D.
Academic Editor
PLOS Pathogens

Alex Andrianopoulos
Section Editor
PLOS Pathogens

Michael Malim
Editor-in-Chief
PLOS Pathogens
orcid.org/0000-0002-7699-2064

****

Thank you for the opportunity to revise and resubmit our manuscript describing the complex roles of Erg251 in *Candida albicans*. Our revised title is: "**Erg251 has complex and pleiotropic effects on ergosterol composition, azole susceptibility, filamentation, and stress response phenotypes**." We include substantial new data that provide mechanistic support for the acquired azole tolerance and stress response phenotypes. We performed comprehensive gas chromatography-mass spectrometry (GC-MS) analysis on the two *ERG251* heterozygous deletion strains, the *ERG251* homozygous deletion strain, and wild-type, both in the absence and presence of fluconazole. This is the first analysis of sterol abundance in both heterozygous and homozygous loss-of-function mutants of *ERG251*, and the first comprehensive comparison of *ERG251* mutant genotypes to wild-type, both in the presence and absence of fluconazole. We were able to include new GC-MS standards that enabled us to confidently identify or exclude the presence of more alternative sterols than any prior study. This new sterol data supports that *ERG251* deletion can cause altered membrane composition, even in the absence of fluconazole. Combining this sterol data with our transcriptional and phenotypic data, we now conclude that the altered membrane composition of *ERG251* mutants causes pleiotropic effects on global gene expression and localization of cell surface proteins that result in the observed phenotypic changes.

Importantly, all prior studies of *C. albicans ERG251* only describe the null mutant, often generated with CRISPR-Cas9 (Xiong et al 2024, Lu et al 2022, Gao et al., 2018). Our work describes a novel mechanism of acquired azole tolerance upon heterozygous loss-of–function of *ERG251*. These azole tolerant mutants remain infectious in a mouse model of systemic infection.

Additionally, we provide rigorous whole genome sequencing and comparative genomic analyses of all the evolved mutants and newly engineered strains, perform comprehensive transcriptomic analyses, and generate extensive quantifiable phenotypic data for this study.

We are grateful for the thoughtful reviews and provide a point-by-point response to all reviewer comments below.

****

Reviewer's Responses to Questions

Part I - Summary
Please use this section to discuss strengths/weaknesses of study, novelty/significance, general execution and scholarship.

Reviewer #1: In this paper, the authors report that under selective pressure in the presence of fluconazole, C. albicans can become drug-tolerant by the acquisition of heterozygous loss-of-function mutations in ERG251, encoding an enzyme in the ergosterol biosynthesis pathway. In combination with certain chromosome aneuploidies these mutations resulted in azole resistance. Strains lacking one ERG251 allele had pleiotropic phenotypes, but in contrast to a homozygous erg251 deletion mutant (which had much stronger phenotypic defects) retained fitness in the absence of the drug and virulence in a mouse infection model. The authors suggest that alterations in sterol biosynthesis and upregulation of the zinc transporter-encoding gene ZRT2 contribute to the acquired azole tolerance of erg251 mutants.

Main comment

1) The authors studied many phenotypic consequences of the ERG251 mutations, but did not investigate how these affected the lipid composition of cellular membranes, which is the expected primary effect of the altered Erg251 activity. The observed phenotypes of the mutants are most likely secondary consequences of an altered membrane structure.

Thank you for the thoughtful and constructive feedback. We agree that additional analysis of lipid composition is critical to the mechanistic understanding of the *ERG251* mutants and subsequent phenotypes in our study. To better understand the mechanisms of drug tolerance and other phenotypes, we performed additional experiments to directly determine the impact of both *ERG251* heterozygous and homozygous deletion on lipid composition in the absence and presence of fluconazole. We performed comprehensive gas chromatography-mass spectrometry (GC-MS)

analysis of sterol accumulation for two *ERG251* heterozygous deletion mutants (*erg251Δ/ERG251* and *ERG251/erg251Δ*), one homozygous deletion mutant *erg251Δ/Δ*, and the SC5314 wild-type control (*ERG251/ERG251*), in two different growth environments: No drug and 1µg/ml fluconazole (**New Fig 6B and 6C**).

In the absence of drug, both heterozygous and homozygous *ERG251* deletion caused significantly reduced ergosterol production, whereas only homozygous deletion of *ERG251* resulted in accumulation of an ergosterol intermediate (**sterol A**, **New Fig 6B and 6C**). We predict that this ergosterol intermediate is 4-methyl episterol, also known as gramisterol or 24-methylene-lophenol (**New Fig S5**). The accumulation of 4-methyl episterol might directly contribute to the growth defect and severe cell membrane sensitivity of the homozygous *ERG251* mutant (**current Fig 3A and New 4C**). In the presence of fluconazole, both heterozygous and homozygous *ERG251* deletion reduced the accumulation of toxic dienol (and toxic dienol intermediates) but increased the accumulation of an alternative sterol that we predicted to be a lanosterol derivative, possibly 24-methylenedihydrolanosterol (**New Fig S5**). We conclude that both the reduced levels of toxic dienol and increased levels of non-toxic alternative sterols support fungal cell growth in the presence of fluconazole and provide mechanistic support for the high azole tolerance of the heterozygous deletion mutants and the increased fitness of *erg251Δ/Δ* at low concentrations of fluconazole (**New Fig 6B and 6C**).

In our initial submission, we showed that *ERG251* deletion led to transcriptional effects on genes encoding cell surface proteins and this gene expression correlated with diverse phenotypes, including drug transport (*ZRT2*), hyphal formation (*HYR1*), and response to stresses (*SOD5, AQY1*). Our new sterol data supports that *ERG251* deletion can cause altered membrane composition, even in the absence of fluconazole. Combining this sterol data with our transcriptional data, we now propose that the altered membrane composition of *ERG251* mutants causes pleiotropic effects on global gene expression and localization of cell surface proteins that result in the observed phenotypic changes.

For a mechanistic understanding of drug tolerance and other mutant phenotypes, knowledge of the underlying primary defect of the heterozygous erg251 mutants would be required (phenotypes and transcriptional changes observed in the erg251 null mutants do not explain the behavior of the heterozygous mutants).

We agree! In the initial submission, we provided extensive comparisons of both heterozygous and homozygous *ERG251* deletions. This is the first study to compare heterozygous and homozygous mutants, and the first to compare the distinct alleles of *ERG251* (A versus B alleles). For example, we generated heterozygous mutants of the A and B alleles of *ERG251* and identified a novel filamentation defect upon deletion of the A allele (*erg251Δ/ERG251*). We performed phenotypic and transcriptomic analyses for both heterozygous deletion mutants and the null mutant and provided transcription-level support for phenotypes observed (**Fig S3**). For example, we found that genes that positively regulate filamentation, including *HYR1* and *HWP1*, and their up-stream transcription factor *SFL2* were all down-regulated upon deletion of the A allele (*erg251Δ/ERG251,* Fig S3A and 5E).

To better understand the primary defect of the *ERG251* heterozygous deletion mutants, we performed additional sterol analysis for both heterozygous mutants as described above. We found that in the absence of drug, heterozygous deletion caused a reduction in ergosterol production **(New Fig 6B and 6C)**. This reduction in ergosterol is likely sufficient to maintain wild-type fitness levels in the absence of fluconazole. Additionally, neither heterozygous mutant had membrane sensitivity like the homozygous deletion mutant, which enabled us to conclude that the accumulation of an ergosterol intermediate, 4-methyl episterol, that was found only in the homozygous deletion mutant is the cause of this membrane sensitivity.

In presence of fluconazole, heterozygous deletion of *ERG251* resulted in decreased levels of toxic dienol and increased levels of the alternative sterol that we predicted to be a lanosterol derivative (**New Fig S5**), providing mechanistic support for the high azole tolerance phenotype of the heterozygous *ERG251* mutants (**New Fig 6B and 6C**). In summary, we used genetic, transcriptional and sterol analysis to support the phenotypes of *ERG251* heterozygous deletion mutants in diverse genetic backgrounds of *C. albicans*. Importantly, all prior studies of *C. albicans ERG251* only

describe the null mutant, often generated with CRISPR-Cas9 (Xiong et al 2024, Lu et al 2022, Gao et al., 2018). Our work describes a novel mechanism of acquired azole tolerance upon heterozygous loss-of–function of *ERG251*.

Interestingly, both reduced and increased ERG251 dosage resulted in azole tolerance (Fig. 1B), but the ERG251-overexpressing strain was not compared with the heterozygous mutants in subsequent experiments and ts unexpected result was not further explored and remains unexplained.

Thank you for suggesting additional discussion of this unexpected result. Indeed, over-expression of *ERG251* also led to increased drug-tolerance (SMG=0.3), but to a lesser extent than *ERG251* heterozygous deletion mutants (SMG=0.6, Fig 1B). Importantly, Erg251 is involved in both the ergosterol biosynthesis pathway and the alternative sterol biosynthesis pathway (**current Fig 6A**). Upon azole treatment, ergosterol biosynthesis is reduced and alternative sterols are increased, leading ultimately to the production of the toxic sterol dienol. Our model is that Erg251 acts as a "rheostat" for both pathways in the presence of azole drugs, helping adjust the amount of flux through the ergosterol pathway while simultaneously controlling the amount of toxic dienol that is produced. Overexpression of *ERG251* likely generates just enough ergosterol to help minimize the effects of azole inhibition of Erg11, supporting a moderately azole tolerant phenotype. Importantly, all *ERG251* point mutations that we identified in our *in vitro* evolution experiments resulted in heterozygous dysfunction of *ERG251* and resulted in high levels of azole tolerance. This overwhelming evidence suggests that reducing Erg251 levels is a more effective strategy for the fungus than overexpression during adaptation to azole drug stress, and more biologically relevant. Therefore, we focused mostly on these loss-of-function mutants in our manuscript.

Reviewer #2:

This manuscript identified a role C. albicans ERG251, a paralog of ERG25, in fluconazole tolerance. An in vitro evolution strategy was used to identify C. albicans strains that are tolerant to fluconazole. Three independent experiments led to the identification of strains with ERG251 heterozygous mutations. The role of ERG251 was confirmed by showing that heterozygous mutants displayed similar fluconazole tolerance. The homozygous erg251/erg251 mutants showed complex phenotypes, including decreased fitness at low initial cell density and increased fitness in the presence of low concentrations of fluconazole. Changes in gene expression detected by RNAseq were used to examine other phenotypes in erg251 mutant strains. These studies showed that the strains were more sensitive to SDS, weakly resistant to H2O2, and displayed a weak hyphal defect. Many ergosterol biosynthesis genes were down-regulated in the erg251D/D strain but the azole tolerance of the heterozygous ERG251 mutant did not appear to be due to changes in ergosterol biosynthesis gene expression. The ZRT2 zinc transporter was upregulated in erg251 mutants, and control studies suggest this may contribute the fluconazole phenotypes. The heterozygous mutants were virulent in a mouse disseminated infection, but the homozygous mutant showed a defect in virulence. Overall, the strength is that these studies discovered a role for ERG251 in promoting fluconazole tolerance in vitro. Weaknesses include the lack of in-depth studies to characterize phenotypes and to better define the role of ERG251 in tolerance.

We appreciate your positive comments and suggestions for improvement.

To better understand the role of *ERG251* in multi-azole tolerance and other phenotypes, we performed additional experiments to directly determine the impact of both *ERG251* heterozygous and homozygous deletion on lipid composition in the absence and presence of fluconazole. We performed comprehensive gas chromatography-mass spectrometry (GC-MS) analysis of sterol accumulation for two *ERG251* heterozygous deletion mutants (*erg251Δ/ERG251* and *ERG251/erg251Δ*), one homozygous deletion mutant *erg251Δ/Δ*, and the SC5314 wild-type control (*ERG251/ERG251*), in two different growth environments: No drug and 1µg/ml fluconazole (**New Fig 6B and 6C**). Results are described above in response to Reviewer 1, and throughout our point-by-point response below.

To better characterize phenotypes of *ERG251* deletion mutants, we performed additional experiments to quantify the phenotypic changes seen in response to cell membrane, osmotic and oxidative stress. We performed growth curve analysis for all *ERG251* mutants in the absence and presence increasing concentrations of each stress (H_2O_2, NaCl, and SDS). This provides a much more comprehensive and quantitative understanding of the phenotypes. By comparing the no drug

control, we quantified relative growth (area under growth curve) at each concentration and determined the minimum concentration that inhibits 20% growth (**New Fig 4C**). Consistent with our previous spot plates results (**moved to Fig S2B**), *erg251Δ/Δ* mutants were more susceptible to osmotic and cell membrane stress but exhibited increased resistance to H_2O_2 (**New Fig 4C**). Additionally, we tested cell wall stressors Calcofluor White and Congo Red and found that cell wall stress does not affect the growth of *ERG251* mutants (heterozygous or homozygous) (**current Fig S2B**).

Reviewer #3:

I really liked all the beginning parts of this paper where the authors told me why I should be interested in ERG251 as a hotspot for studying antibiotic resistance. It was novel and very convincing. Then we come to the experiments characterizing the single and double knockout mutants of erg251. The results here are interesting, bordering on fascinating, but I found it difficult to consider them in the proper context. Fig 6A did not help in this regard. I would like to see a clear presentation of where you think Erg25p and 251p fit into biosynthesis. In reading Lu et al (2023), they had Erg25p in the main sterol biosynthesis pathway and Erg251p in the alternative pathway, but you seem to be avoiding such a clear distinction. I think this portion of the text would be more easily understood if accompanied by a diagram presenting your best thoughts on how the 251p fits into biosynthesis.

Thank you for your appreciation of the work and helpful feedback. We agree that a clearer distinction between Erg25 and Erg251 in our diagram is helpful for understanding their roles in the ergosterol and alternative pathways. In our revised Fig 6A, we place both *ERG25* and *ERG251* on the late ergosterol biosynthesis pathway, but only *ERG251* in the alternative sterol biosynthesis pathway (**Current Fig 6A**). Evidence for this placement comes from our new analysis of the sterol composition changes that occur upon *ERG251* deletion. As described above for Reviewer 1 & 2, we performed comprehensive gas chromatography-mass spectrometry (GC-MS) analysis of sterol accumulation for two *ERG251* heterozygous deletion mutants (*erg251Δ/ERG251* and *ERG251/erg251Δ*), one homozygous deletion mutant *erg251Δ/Δ*, and the SC5314 wild-type control (*ERG251/ERG251*), in two different growth environments: No drug and 1μg/ml fluconazole (**New Fig 6B and 6C**). In the absence of fluconazole, both heterozygous and homozygous deletion of *ERG251* caused decreased accumulation of ergosterol (**New Fig 6B and 6C**). Furthermore, we provide evidence that *ERG25* transcripts are increased in the absence of *ERG251* (current Fig S4A and S6). The upregulation of *ERG25* can compensate for the loss of *ERG251* during ergosterol biosynthesis, resulting in sustained (albeit reduced) ergosterol production. Surprisingly, in the absence of fluconazole, low levels of the toxic dienol (the end product of the alternate sterol pathway) are detected in wild-type and heterozygous *ERG251* deletion strains. This toxic dienol is not detected in the homozygous *ERG251* deletion mutant, in the absence of fluconazole (**current Fig 6C**). Erg251 is therefore the major active C-4 sterol methyl oxidase in the alternate sterol pathway under normal growth conditions.

In the presence of fluconazole, both the heterozygous and homozygous *ERG251* deletion mutants had decreased levels of the toxic dienol and increased levels of an alternative sterol at 25.1 min retention time with a similar spectra profile as lanosterol. Furthermore, we found that *ERG25* deletion had no effect on azole drug susceptibility, similar to Lu *et al* 2022. Therefore, we conclude that *ERG251* is the major active C-4 sterol methyl oxidase that regulates azole susceptibility. Consistent with Lu et al 2022, we find that *ERG251* plays a vital role in the alternative pathway (Lu et al 2022).

Similarly, Lu et al stated that the erg251 double null mutant failed to grow in the presence of fluconazole (their Fig 4A-C) while you have the double 251p mutant presenting a fitness advantage at low fluconazole concentrations and a fitness cost at high fluconazole concentrations. Is this strictly a difference in concentrations chosen, or does it present something more fundamental? To me, such differences suggest multiple targets for the azole inhibitors.

Thank you for the thoughtful and constructive feedback. First, we would like to highlight that our fluconazole growth curve results are consistent with the fluconazole microbroth dilution assay presented in Lu *et al.* for the *erg251Δ/Δ* null mutants. We both show the *erg251Δ/Δ* null mutant has a growth **benefit** in low fluconazole concentrations and a **defect** in high fluconazole concentrations.

However, they did not address the growth benefit of the *erg251*Δ/Δ mutant in their paper, possibly because their agar plate assay in high fluconazole had such a strong growth defect.

Here, we compare our results with theirs for additional clarification:
In our Fig 3C (provided below, right), we compared growth across a broad range of fluconazole concentrations (0.25 - 256 µg/ml fluconazole) in a 96-well plate assay with constant shaking. We report that the *erg251*Δ/Δ null mutant has increased growth (indicated by area under growth curve) in 0.25, 0.5 and 1 ug/ml fluconazole, but not at higher concentrations of fluconazole. We also quantified relative fitness using a head-to-head competition assay between the *erg251*Δ/Δ null mutant and a fluorescently labelled wild-type strain and conclude that the *erg251*Δ/Δ null mutant had increased fitness in the presence of 1ug/ml fluconazole, and decreased fitness in rich medium relative to the wild-type strain (our Fig 3E).

Similarly, Lu *et al.* report higher relative growth of the *erg251*Δ/Δ null mutant in presence of low concentrations of fluconazole compared to the no drug control in a microbroth dilution assay (Lu et al 2023 Fig 4C, provided below, left). Therefore, our data are consistent that low concentrations of fluconazole (≤1µg/ml) can increase the growth *erg251*Δ/Δ*,* while in the presence of high concentrations of fluconazole the growth of *erg251*Δ/Δ remains poor.

Fig 4C. Lu et al 2023 | Fig 3C. This manuscript

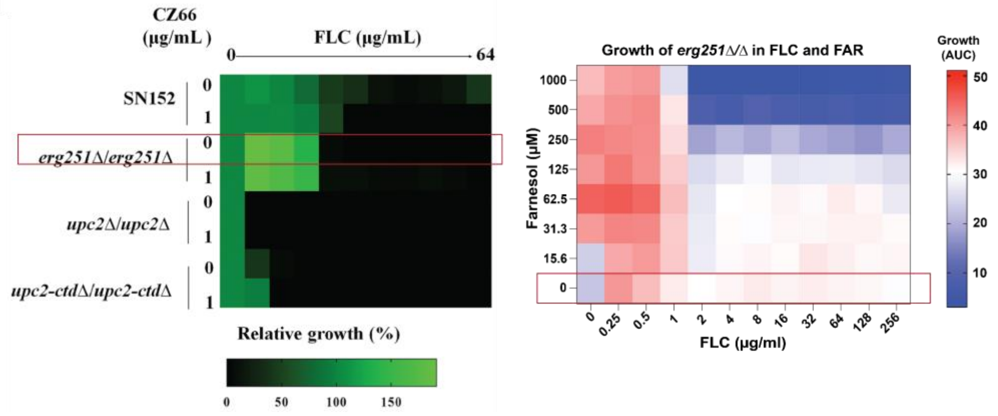

Both the Erg25p and 251p are C-4 sterol methyl oxidases. Candida albicans and Aspergillus fumigatus have two such enzymes while S. cerevisiae and the ergosterol containing algae Chlorella (Voshall et al 2021) and Chlamydomonas (Brumfield et al 2017) have only one. There are two methyl groups on the C4 position of lanosterol which need to be removed. Perhaps there is a division of labor. Voshall et al examined the sterols which were produced by Chlorella following inhibition by either ketoconazole or clotrimazole (fluconazole was not inhibitory). The relevant feature for your paper was that identifying the unusual or overflow sterols produced following these antibiotic treatments strongly suggested that four different sterol precursors were available as substrates for the single C4-sterol methyl oxidase. Could Erg25p and 251p exhibit different substrate preferences? Please note that I am not suggesting detailed sterol compositions for each of your mutants following antibiotic treatment. Those experiments might be desirable in the long term but highly impractical in the short term. I'm merely seeking clarification on your thinking on the respective roles of Erg25p and Erg251p.

Thank you for these great ideas. We appreciate that there might be differences in substrate preference between *ERG251* and *ERG25*. While not requested, we opted to perform extensive sterol analysis of wild-type and *ERG251* heterozygous and homozygous mutants and are pleased to report those results here and in the revised manuscript. The additional data also allowed for additional clarification of the roles of Erg25p and Erg251p as discussed in the manuscript and below. Overall, the potential difference in substrate preferences might explain why Erg251p is the primary C-4-sterol methyl oxidase for the alternative sterol pathway. We now include this idea in the discussion section and briefly here.

In the absence of drug, heterozygous and homozygous deletion of *ERG251* results in reduced ergosterol production (**New Fig 6B and C**). Additionally, Xiong et al. found ectopic over-expression of *ERG25* can partially recover the ergosterol production in the *erg251*Δ/Δ mutant (Xiong et al. 2024). We conclude that *ERG25* can partially compensate for the loss of *ERG251* in ergosterol biosynthesis. Our data suggest that *ERG251* and *ERG25* both utilize 4,4-dimethyl zymosterol as a

substrate towards ergosterol production. As evidence, we did not detect accumulation of 4,4-dimethyl zymosterol, the common substrate C-4 sterol methyl oxidases, in any *ERG251* deletion mutant (heterozygous or homozygous). Interestingly, homozygous deletion of *ERG251* resulted in accumulation of an ergosterol intermediate 4-methyl-episterol, also known as gramisterol or 24-methylene-lophenol and this ergosterol intermediate was absent from the heterozygous deletion mutants under both growth conditions (**New Fig 6B and C**). This suggests that *ERG251* has a substrate preference for 4-methyl-episterol during ergosterol production. Similar to what was found in *Chlorella sorokiniana*, enzymes in the ergosterol biosynthesis pathway can have multiple possible substrates to support flux through ergosterol production upon azole inhibition (Voshall et al 2021).

In the presence of low levels of fluconazole, *ERG251* deletion mutants had accumulation of an alternative sterol that is predicted to be 24-methylenedihydrolanosterol. This alternative sterol is different from eburicol, another common substrate for C-4 sterol methyl oxidase, that Lu et al. 2022 predicted to accumulate in the erg251 null mutant in the presence of high levels of fluconazole. Importantly, we included an eburicol standard in our GC-MS analysis and do not detect any eburicol in our strains. This suggests that there might be different substrates for *ERG251* in the alternative pathway and that azole concentration (degree in which the ergosterol biosynthesis pathway is inhibited) might impact the substrate preference.

Reviewer #4:

The manuscript by Zhou et al describes in detail the discovery that growth of C. albicans strains in fluconazole can lead to the development of single allele ERG251 mutations that increase fluconazole tolerance. When these heterozygous ERG251 mutations were combined with spontaneous aneuploidies in chromosomes 3 or 6, they also saw full resistance to fluconazole. In addition, they characterized the impacts of heterozygous ERG251 mutations on either or both alleles for their impacts on growth rate, global gene transcription, hyphal growth, cell wall sensitivities, and virulence in mice. Their findings implicated ERG251 in impacting recovery from lag phase at low density growth through affecting farnesol. They found that one allele of ERG251 was more effective than the other for several phenotypes like hyphal growth and transcription of other ERG genes. They also found that the ZRT2 zinc transporter's transcription was impacted by ERG251 and could affect azole sensitivity of wild-type when overexpressed. Finally, the erg251ΔΔ mutant was diminished in virulence, but the heterozygotes were not.

Overall, I found this manuscript to have a wide breadth of coverage of many different phenotypes, but several at a mostly superficial level. There was not a clear model for how Erg251 affects these many different phenotypes such as transcription or hyphal growth.

We appreciate the feedback and appreciation for the breadth of phenotypes analyzed. We significantly revised the latter half of the manuscript to provide a clearer model for how Erg251 affects diverse cellular phenotypes including drug susceptibility, filamentation, and stress response. The addition of new, comprehensive sterol analysis, both in the absence and presence of azole drug stress, enabled us to solidify our model. The sterol results are described above in response to Reviewer 1 and 3, and throughout our point-by-point response below. ***ERG251* deletion caused significantly altered sterol composition, even in the absence of azole exposure.** In our initial submission, we showed that *ERG251* deletion led to transcriptional effects on genes encoding cell surface proteins and this gene expression correlated with diverse phenotypes, including drug transport (*ZRT2*), hyphal formation (*HYR1*), and response to stresses (*SOD5, AQY1*). Our new sterol data supports that *ERG251* deletion can cause altered membrane composition, even in the absence of fluconazole. Combining this sterol data with our transcriptional data, we now propose that the altered membrane composition of *ERG251* mutants causes pleiotropic effects on global gene expression and localization of cell surface proteins that result in the observed phenotypic changes.

Part II – Major Issues: Key Experiments Required for Acceptance
Please use this section to detail the key new experiments or modifications of existing experiments that should be <u>absolutely</u> required to validate study conclusions.

Generally, there should be no more than 3 such required experiments or major modifications for a "Major

Revision" recommendation. If more than 3 experiments are necessary to validate the study conclusions, then you are encouraged to recommend "Reject".

Reviewer #1: Other comments

2) To test whether the slight upregulation of ZRT2 contributed to the fluconazole tolerance of erg251 mutants, the authors overexpressed ZRT2 in a wild-type strain (overexpression levels should be given and compared to those in erg251 mutants). To more directly address the question and support the conclusion stated in the paragraph title (lines 560-561), one could test if ZRT2 deletion (possibly only one allele if this reduces expression levels to those in the wild type) in erg251 mutants reverts tolerance.

We appreciate these suggestions.

To better delineate the effects of *ZRT2* on *ERG251*-mediated azole tolerance, we deleted one copy of *ZRT2* from the *ERG251* heterozygous mutant background. Both independent transformants exhibited reduced fluconazole tolerance (SMG = 0.45-0.49) compared to the *ERG251* heterozygous mutant (SMG = 0.6) (**New Fig 7D**). We conclude that *ZRT2* is directly contributing to the high azole tolerance seen in the *ERG251* heterozygous mutant, however it is not the only contributor. Therefore, the *ERG251*-mediated azole tolerance is a combination of the effects of *ZRT2* and sterol composition changes (**New Fig 6B and 6C**)

Additionally, we quantified the expression levels of *ZRT2* in the *ZRT2* overexpression strain using RT-qPCR and compared it to the expression of *ZRT2* in both *ERG251* heterozygous deletion mutants. The increase in *ZRT2* expression is similar between the tetO-*ZRT2* overexpression strains and the heterozygous *ERG251* deletion strains in the presence of FLC (1.5 to 1.6-fold increase relative to the wild-type, **New Fig 7C**).

3) Is the allele-specific effect of ERG251 on filamentation in the SC5314 background also seen in the evolved strains (SN152 and BWP17 are derivatives of SC5314), i.e. does Evolved 3.2 behave like the heterozygous mutant in which allele B was deleted, and do AMS5617/5618, AMS5622/5623/5624, and AMS5625/5626 behave like the heterozygous mutant lacking allele A? Furthermore, did introduction of the loss-of-function mutations into alleles A or B of strain SC5314 (lines 194-198 and Fig. 1A) have the same allele-specific effect on filamentation?

We appreciate this question. As suggested, we tested the filamentation phenotype for both evolved and engineered strains with *ERG251* point mutations in either the A or B allele.

For example, the effect of point mutation on the *ERG251*-A allele was analyzed in the evolved strains AMS5625 (*ERG251*^{W265G}/*ERG251*) and AMS5626 (*ERG251*^{W265G}/*ERG251*), and in the engineered strain containing the same point mutation (*ERG251*^{W265G}/*ERG251*). The effect of point mutation on the *ERG251*-B allele was similarly analyzed in the evolved strain AMS5730 (*ERG251*/*ERG251*^{*322Y}) and in the engineered strain containing the same point mutation (*ERG251*/*ERG251*^{*322Y}) (**New Fig S3E**).

Loss-of-function mutation on the *ERG251-A* allele caused reduced filamentation while mutation on the *ERG251-B* allele resulted in almost no change in filamentation (**New Fig S3E**). In summary, the allele-specific effect of *ERG251* on filamentation is seen both for evolved and engineered *ERG251* point mutants.

Revisions in manuscript are as follows: “Similar filamentation defects were also observed for both evolved and engineered strains with *ERG251* loss-function point mutation on A allele (*ERG251*^{W265G}/*ERG251*), but not for strains with point mutation on B allele (*ERG251*/*ERG251*^{*322Y}) (Fig S3E).”

Reviewer #2:

1. The legends to Fig. 1 and 2 state that each bar represents the average of three technical replicates. Were these studies repeated in independent experiments? No error bars are shown.

Thank you for suggesting additional clarification. In current Fig 1, Fig 2, Fig 7, and Fig S4, error bars are now added. To clarify, these figures describe a microwell broth dilution assay to determine drug susceptibility phenotypes: the MIC (minimum inhibitory concentration) and SMG (supra-MIC growth). The MIC_{50} of each strain was determined to be the drug concentration at which ≥50% of growth was inhibited when compared with the no-drug control at 24 hrs post-inoculation. The supra-MIC growth (SMG) was measured as the average growth above (OD_{600}) the MIC_{50} when standardized to the no-drug control at 48 hrs post-inoculation. For each strain we did three technical replicates in all figures and each bar represents the average of all three replicates of the same strain. We applied error bars to indicate the standard error of the mean (SEM) for SMG. However, because the MIC_{50} is a categorical variable (the drug concentration that inhibits growth by 50%) then no error bars can be presented when the MIC_{50} is consistent across replicates.

2. Fig. 3A. Is this a representative curve? Average of three assays? What were the lag phases for the three assays? Do erg251 deletion mutants grow better in conditioned medium?

Thank you for these questions. Additional information was added to Fig 3A and its legend. Fig 3A is the average growth curve of three technical replicates with the standard error of the mean now provided. As suggested, an analysis of each growth phase (including lag phase) is also included **in New Fig 3B**. The *ERG251* homozygous deletion mutant exhibited a similar lag phase as the wild-type, however the lag phase was shorter with increased initial cell density (OD = 0.005 and 0.01 compared to 0.0001).

As for conditioned medium, our preliminary experiments identified that conditioned medium improved the growth of the *ERG251* deletion mutants. This observation, in combination with the cell density dependent growth differences, led us to focus on the role of the quorum sensing molecule farnesol. We found that a range of farnesol concentrations can improve growth of the homozygous *ERG251* deletion mutant in the absence of fluconazole and even in low concentrations of fluconazole (Fig 3C, **Y-axis**). However, high concentrations of both farnesol and fluconazole have fungicidal effects when *ERG251* is absent.

3. Figure 4C is not supported by strong data. (i) A slight increase in sensitivity to SDS could be due to many reasons, such as altered membrane lipids. It is not specifically indicative of altered cell wall. (ii) Increased resistance to oxidative stress is not well supported. There appears to be a very weak effect in a spot assay. This should be quantified. The magnitude of the resistance does not seem very significant. Was it reproducible?

Thank you for suggesting additional clarification and quantification of the spot plate phenotypes.

(i) We agree that SDS causes cell membrane stress, not cell wall stress, and the text was changed accordingly. Additionally, to better understand the susceptibility of *ERG251* deletion mutants to cell wall stress, we tested the growth of all *ERG251* mutants on Calcofluor White and Congo Red. Cell wall stress does not affect the growth of *ERG251* mutants (heterozygous or homozygous) (**current Fig S2B**).

(ii) To better quantify the phenotypic changes seen in response to cell membrane, osmotic and oxidative stress, we performed additional experiments and analyses. We performed growth curve analysis for all *ERG251* mutants in the absence and presence increasing concentrations of each stress (H_2O_2, NaCl, and SDS). By comparing the no drug control, we quantified relative growth (area under growth curve) at each concentration and determined the minimum concentration that inhibits 20% growth (**New Fig 4C**), and plotted these values for the wild-type and mutant strains. Consistent with the original spot plates (**moved to Fig S2B**), *erg251*Δ/Δ is more susceptible to osmotic and cell membrane stress but exhibited increased resistance to H_2O_2 (**New Fig 4C**). This provides a much more comprehensive and quantitative understanding of the phenotypes.

4. Figure 5. There appears to be a slight defect in hyphal growth for ERG251-A deletion and a little stronger defect for erg251D/D mutant. However, it is not clear that this is significant. Only limited characterization

was presented. Also, it was not clear that there was a defect in vivo. No analysis of hyphal growth in vivo was presented.

Thank you for these comments. The filamentation assay we used here is a well-adopted method to quantify *Candida albicans* filamentation *in vitro* (Huang et al 2015, Cravener et al 2023, Kakade et al 2023, Glazier et al 2023). We quantified the population fractions of cells in the yeast, hyphal and pseudohyphal states for three independent biological replicates. 150 to 500 cells were counted for each biological replicate. Among all three biological replicates, the *ERG251* homozygous deletion mutant exhibited a statistically significant decrease in the fraction of hyphal cells (Fig 5B, *P≤ 0.05).

While we agree it would have been nice to compare the filamentation profiles of the *ERG251* mutants *in vivo*, we did not obtain histology data from our mouse experiments, and acquiring these data would require significant additional time, effort, and animals. Additionally, recent examples from the literature indicate that a filamentation defect *in vitro* does not indicate that the same phenotype will be observed *in vivo* (Glazier et al 2023). Multiple host stimuli can induce filamentation in *C. albicans*, and in some cases these combined stimuli are sufficient to overcome the filamentation defects observed *in vitro*.

5. Figure 6 is very descriptive. It shows that ERG gene expression is altered, but it is not clear if this is significant.

Thank you for suggesting additional clarification. In the initial submission, Fig 6 contained the expression level changes (log2-fold change) for genes involved in ergosterol biosynthesis pathways comparing the *ERG251* deletion mutants and wild-type across environments. In the current manuscript, we moved these log2-fold change heat maps into supplementary figures (**current Fig S4A and B**) and provide significance values as follows: In **current Fig S4A**, we used two asterisks to indicate genes that had significant expression level changes (adjusted *p*-value < 0.05) in *erg251Δ/Δ* relative to wild-type in both YPAD (S2 Table) and YPAD+1µg/ml (S11 Table) fluconazole conditions, and one asterisk to indicate *ERG6* had significant expression changes (adjusted *p*-value < 0.05) in *erg251Δ/Δ* related to *ERG251/ERG251* only in YPAD+1µg/ml fluconazole conditions (S11 Table). In **current Fig S4B**, we again used one asterisk to indicate genes that had significant expression changes (adjusted *p*-value < 0.05) in *erg251Δ/Δ* in YPAD relative to *erg251Δ/Δ* in YPAD+1µg/ml fluconazole conditions (S15 Table) (methods, RNA-Seq data analysis).

Revisions are made below:
Current Fig S4A: "Two asterisks indicate the expression change is significant (adjusted p-value < 0.05) in *erg251Δ/Δ* relative to *ERG251/ERG251* in both YPAD (S2 Table) and YPAD+1µg/ml FLC (S11 Table) conditions. One asterisk: *ERG6* expression level change is significant (adjusted p-value < 0.05) in *erg251Δ/Δ* relative to *ERG251/ERG251* only in YPAD+1µg/ml FLC (S11 Table) condition."
Current Fig S4B:" One asterisk indicates the expression change is significant (adjusted *p*-value < 0.05) in *erg251Δ/Δ* in YPAD relative to *erg251Δ/Δ* in YPAD+1µg/ml fluconazole conditions (S15 Table)."

6. Figure 7. Lines 591-596. The conclusion that altered ZRT2 expression contributes to fluconazole tolerance of erg251/ERG251 mutants is not supported by strong data. The TET-promoter strain showed increased tolerance to fluconazole, but it was not clear what was the level of ZRT2 expression in the Tet-ZRT2 strain. Also, it was not clear how the authors concluded that the ZRT2 effect is distinct from distinct from the ATP-dependent drug efflux pumps such as CDR1.

Thank you for these suggestions. As mentioned above, several reviewers had similar suggestions. We engineered additional strains and performed quantitative PCR to address all reviewers' points.

To better delineate the effects of *ZRT2* on *ERG251*-mediated azole tolerance, we deleted one copy of *ZRT2* from the *ERG251* heterozygous mutant background. Both independent transformants exhibited reduced fluconazole tolerance (SMG = 0.45-0.49) compared to the *ERG251* heterozygous mutant (SMG = 0.6) (**New Fig 7D**). We conclude that *ZRT2* is directly contributing to the high azole tolerance seen in the *ERG251* heterozygous mutant, however it is not the only contributor.

Therefore, the *ERG251*-mediated azole tolerance is a combination of the effects of *ZRT2* and sterol composition changes (**New Fig 6B and 6C**).

Revision made as below:

“To delineate the role of Zrt2 in Erg251-mediate azole tolerance, we engineered additional strains and quantified drug susceptibility. Overexpression of *ZRT2* in the wild-type SC5314 background resulted in an ~1.6-fold increase in mRNA expression relative to wild-type in the presence of FLC (Fig 7C). Overexpression of *ZRT2* caused increased FLC tolerance (SMG = 0.24-0.25) relative to wild-type, however less tolerance than the *ERG251* heterozygous deletion mutants (SMG = 0.6) (Fig 7D). To test if the Zrt2 directly contributes to the high tolerance observed in *ERG251* heterozygous deletion mutants, we deleted a single copy of *ZRT2* from the *erg251Δ/ERG251* background (Fig 7D). Both independent transformants exhibited reduced FLC tolerance (SMG = 0.45-0.49) compared to the *ERG251* heterozygous deletion mutant (SMG = 0.6) (Fig 7D). Taken together, we conclude that Zrt2 directly contributes to *ERG251*-mediated azole tolerance together with sterol composition changes.”

We agree that the statement about ATP-dependent drug efflux mechanisms was confusing. This statement was removed in the revised discussion.

7. Figure 8. Mouse virulence. It would have been interesting to see if there was a difference in ability of fluconazole to prevent lethal infection. Perhaps a mixed infection to see if the heterozygotes have a better ability to survive? Some type of experiment like that would have strengthened the conclusions.

We appreciate the suggestions for additional mouse experiments. However, we respectfully disagree that the additional animal experiments will substantially increase scientific value of the study. Our current study has already utilized a large number of animals. Additional drug treatment experiments would require us to kill significantly more animals, as we would need to perform preliminary experiments to get the drug concentration (dosage schedule) and *in vivo* competition studies optimized, all before testing the effects of fluconazole. The resources required for these additional animal experiments (time, funding, and personnel) are substantial. Given the comprehensive nature of our current dataset, the incremental knowledge gained from additional experiments is unlikely to justify these costs. We appreciate your understanding and consideration of these points and are open to discussing any specific aspects of our data that may still be of concern.

8. The Discussion section contained a lot of speculation about minor effects that could be shortened. Lines 755 -765. Not proven ZRT2 was overexpressed, or expressed at the level seen in the erg251 mutant.

Thanks for these suggestions. We shortened this discussion section as suggested. Additionally, we quantified the expression levels of *ZRT2* in the tetO-*ZRT2* overexpression strains compared to the expression of *ZRT2* in both *ERG251* heterozygous deletion mutants. The increase in *ZRT2* expression is similar between the tetO-*ZRT2* overexpression strains and the heterozygous *ERG251* deletion strains in the presence of FLC (1.5 to 1.6-fold increase relative to the wild-type, **New Fig 7C**).

Reviewer #3:

None

Reviewer #4:

1. For example, the overexpression study with ZRT2 implicates it in being an effector of ERG251 fluconazole sensitivity, but additional work would be needed to draw this conclusion definitively. Overexpression of ZRT2 clearly impacts this phenotype, but it could be a parallel pathway rather than a direct linear effect related to ERG251.

Thank you for these suggestions. As mentioned above, several reviewers had similar suggestions. To better delineate the effects of *ZRT2* on *ERG251*-mediated azole tolerance, we deleted one copy of *ZRT2* from the *ERG251* heterozygous mutant background. Both independent transformants exhibited reduced fluconazole tolerance (SMG = 0.45-0.49) compared to the *ERG251* heterozygous mutants (SMG = 0.6) (**New Fig 7D**). We conclude that *ZRT2* is directly contributing to the high azole tolerance seen in the *ERG251* heterozygous mutant, however it is not the only contributor. Therefore, the *ERG251*-mediated azole tolerance is a combination of the effects of *ZRT2* and other sterol composition changes (**New Fig 6B and 6C**).

2. The localization of ERG251-GFP needs to be better controlled. Were these alleles able to complement an erg251ΔΔ mutant? Colocalization with a known ER marker needs to be done make the conclusion of ER localization

We appreciate these suggestions. To clarify, we tagged Erg251-A or Erg251-B with GFP in the wild-type background by fusing *GFP* at the C-terminal of *ERG251-A* or *ERG251-B*. Independent complementation of *erg251Δ/Δ* with either the *ERG251-A* or *ERG251-B* allele was performed and correct transformants were validated by whole genome sequencing. As indicated in Fig 3G, Fig 5 A&B, and Fig 8A, complemented strains all rescued the severe defects of the *erg251Δ/Δ* null mutant and had similar phenotypes as the corresponding heterozygous deletion mutants.

To show the colocalization of Erg251 and ER, we applied a commercial ER tracker to Erg251-A-GFP and Erg251-B-GFP strains and found colocalization of Erg251 with ER in yeast cells (**current Fig S3G**). However, this commercial ER tracker was not detectable in hyphae using the same protocol, which is likely caused by the membrane dynamic differences between yeast and hyphae (Benhamou et al. 2018, Pulver et al. 2013, Jones et al. 2010).

3. The conclusion drawn in lines 222-225: “We found that ERG251 -driven azole tolerance was independent of drug efflux pumps as indicated by no increase in the rate of efflux of R6G for ERG251 heterozygous deletion mutants compared to ERG251/ERG251 (SC5314) during the exposure to fluconazole (Fig 1C).” is not consistent with the data shown in Fig 1C where it appears that mutants are progressively less able to excrete the drug than the wild-type.

Thank you for catching this error. We agree that mutants had a lower efflux rate of R6G.

Revisions are as follows:
“We found that *ERG251*-mediated azole tolerance was independent of drug efflux pumps as indicated by a small decrease in the rate of efflux of R6G for *ERG251* heterozygous deletion mutants compared to *ERG251/ERG251* (SC5314) during the exposure to fluconazole (Fig 1C).”

4. In Figure 4, SDS is a better measure of membrane stress, but not cell wall stress. Specific stressors of the cell wall such as echinocandins, calcofluor white, and Congo Red should be tested.

Thank you, we agree that SDS is a membrane stress and revised the text accordingly. As suggested, we tested additional cell wall stressors, including calcofluor white and Congo Red. None of the *ERG251* deletion mutants exhibited growth defects in the presence of cell wall stressors (Fig S2B).

Revisions are made below:
“Compared to wild-type (*ERG251/ERG251*), *erg251Δ/Δ* exhibited no change in response to increased temperature (37°C) or cell wall stressors (Calcofluor White and Congo Red) (Fig S2B). In contrast, erg251Δ/Δ exhibited detectable phenotypes in response to cell membrane, osmotic and oxidative stress (Fig S2B). To quantify these effects, we performed growth curve analysis in the absence and presence of increasing concentrations of H2O2, NaCl, and SDS, and calculated the minimum concentration that inhibited growth by 20% relative to no stress. The erg251Δ/Δ mutant was more susceptible to osmotic (NaCl, 1.4-fold decrease) and cell membrane (SDS, 10-fold decrease) stress, but exhibited increased resistance to H2O2 (1.5-fold increase) relative to wild-type (New Fig 4C and Fig S2B).

Part III – Minor Issues: Editorial and Data Presentation Modifications
Please use this section for editorial suggestions as well as relatively minor modifications of existing data that would enhance clarity.

Reviewer #1:

4) The mutation *322Y (line 174 and Table 1) is incorrectly described as *321Y in line 197 and Fig. 1A.

Thank you for catching this. The correct genotype *322Y is now corrected throughout.

5) I did not find the mysterious positive control strain, which is mentioned on several occasions (lines 229, 252, 1098), in Table S1. Its name and a reference should be given.

Thank you for pointing this out! We regret that information about this positive control was missing from the original manuscript. The positive control is a well-characterized fluconazole resistant clinical isolate C17/12-99 that has increased expression of *ERG11*, MDR1, *CDR1* and *CDR2* (White et al 2002; Rogers and Barker 2003). We added the isolate name, information, and citations to the main text and S1 Table.

For example: "Addition of an Hsp90 inhibitor (radicicol, 2.5μM) to assays measuring azole resistance (MIC_{50}) and tolerance (SMG) blocked the acquired azole tolerance of *ERG251* heterozygous deletion mutants. However, radicicol did not inhibit growth of a well-characterized FLC resistant clinical isolate with increased expression of *ERG11*, *MDR1*, *CDR1* and *CDR2* (C17/12-99, Fig 1D, and S1 Table) (White et al 2002, Rogers and Barker 2003)."

The authors state that radicicol did not inhibit growth of this strain (lines 226-229), but Fig. 1D shows that growth was inhibited by 75%.

To clarify the growth inhibition, the fluconazole resistant clinical isolate C17/12-99 (positive control) had an MIC at 128 μg/ml FLC, both with and without radicicol treatment (Fig 1D). More importantly, as indicated in Fig S2A this fluconazole resistant isolate (positive control) had no change in cell viability with or without radicicol treatment (Fig S2A). In contrast, *ERG251* heterozygous mutants exhibited no viable cells above MIC concentration with radicicol treatment (Fig S2A). Taken together, this supports that radicicol did not reduce the MIC or the cell viability of this fluconazole resistant isolate.

Revision as made below:
"Radicicol did not reduce the MIC or the cell viability of a well-characterized FLC resistant clinical isolate with increased expression of *ERG11*, *MDR1*, *CDR1* and *CDR2* (C17/12-99, Fig 1D, and S1 Table) (White et al. 2002; Rogers and Barker 2003)."

6) The statement in lines 679-682 is not correct. Although mutations in ERG11 and UPC2 that cause increased azole resistance are indeed often found in both alleles, strains containing such mutations in only one allele have also been described, and the mutations confer increased drug resistance also in heterozygous strains (albeit at a lower level).

We agree. Heterozygous mutations of *ERG11* and *UPC2* that cause increased azole resistance have been described. We modified our statement to focus on homozygous mutations that result in high MIC values and include additional citations that reference the heterozygous point mutations as well.

Revisions are made as below:
"For example, point mutations in ergosterol-related genes like *ERG11* and *UPC2* that cause drug resistance in *Candida* species are frequently homozygous in diploid organisms and result in higher MIC than heterozygous mutations (Flowers et al. 2012; Flowers et al. 2015; Rybak Jeffrey M. et al. 2021; Burrack et al. 2022; Li et al. 2024; Dunkel et al. 2008)."

7) The authors point out in the abstract (lines 30-31) that this report provides the first known example of point mutations causing azole tolerance in C. albicans, but similar findings are apparently reported in an

already submitted manuscript by the same group (Zhou et al. 2024, under review; see lines 141-143).

Sorry for the confusion. We originally cited Zhou et al 2024 (in revision) because it described the methodology for a large parallel *in vitro* evolution experiment where one of the *ERG251 de novo* mutations was identified by whole genome sequencing. Because this mutation was not analyzed in the cited study, we have removed this citation to eliminate additional confusion and to highlight the novelty of the current study.

8) The authors should check the text for correct expressions. For example, mutations cannot cause an infection (lines 617-619); it was the mutants that remained infectious. There are additional instances of formally incorrect wording.

Thank you for catching these errors in wording. We made corrections throughout the text, including the lines suggested:
"Taken together, this indicates that mutants with homozygous deletion of *ERG251* have attenuated virulence, which supports the importance of *ERG251* in varied cellular responses essential for pathogenicity. However, the azole tolerant mutants with a single allele dysfunction of *ERG251* remained infectious."

Reviewer #2:

Page 7 Intro. Mention that deletion of erg3 rescues lethality of erg11 deletion

Thank you for this suggestion. Revisions are as follows:
"ERG3 inactivation causes reduced toxic dienol and instead results in accumulation of 14α-methylfecosterol which supports growth in presence of azoles despite altered membrane composition (Kelly et al. 1997; Martel et al. 2010; Branco et al. 2017). *ERG3* inactivation also rescues the lethality of ERG11 deletion mutants in multiple species (Bard et al. 1993; Sanglard et al. 2003; Kelly et al. 1995)."

Show WT controls in Table 1

Thank you for this suggestion. The progenitors for each independent evolution experiment are now included in Table 1.

Fig. 1D. Explain the rational for testing the Hsp90 inhibitor.

Thank you for this request. We rearranged this paragraph and added rationale and citations for testing the inhibition of Hsp90 immediately before the description of the data. Revisions are made below:

"Hsp90 is a molecular chaperone and an important mediator for drug-tolerance and stress response (Kim et al 2019, Robbins et al 2023). We found that *ERG251*-mediated tolerance depends on Hsp90 function. Addition of an Hsp90 inhibitor (radicicol, 2.5μM) to assays measuring azole resistance (MIC_{50}) and tolerance (SMG) blocked the acquired azole tolerance of *ERG251* heterozygous deletion mutants. Radicicol did not reduce the MIC or the cell viability of a well-characterized FLC resistant clinical isolate with increased expression of *ERG11*, *MDR1*, *CDR1* and *CDR2* (C17/12-99, Fig 1D, and S1 Table) (White et al. 2002; Rogers and Barker 2003)."

Line 419. The increased resistance to H2O2 was very weak, and was not quantified.

Thank you for suggesting additional quantification and analyses of this phenotypic data.
In general, we find that spot plate assays are often insufficient for determining the level and effect of environmental stress. Therefore, to provide better quantification for the susceptibility changes seen in response to oxidative stress, osmotic and membrane stress, we performed 96-well plate growth curve analysis for all *ERG251* mutants in the absence and presence of increasing concentrations of each stress (H_2O_2, NaCl, and SDS). This provided a much more comprehensive and quantitative understanding of the phenotypes. We quantified relative growth (area under growth curve) at each concentration and determined the minimum concentration that inhibits 20% growth (**New Fig 4C**).

Consistent with our previous finding using spot plate assays (**moved to Fig S2B**), the *erg251Δ/Δ* strain was more resistant to H_2O_2 (**New Fig 4C and Fig S2B**).

Line 727. Changes in cell wall sensitivity is too strong of a statement. The mutant cells showed a modest increase in sensitivity to SDS. The detergent SDS is most likely to affect the plasma membrane, and is not likely to affect the cell wall directly. Thus, the effects of SDS could be due to altered plasma membrane lipids.

Thank you. We agree that "increased cell wall sensitivity of *erg251Δ/Δ"* was incorrect and removed this line from the text. We also agree that the effect of SDS is due to the altered plasma membrane lipids. Our new sterol analysis supports that the SDS sensitive *erg251Δ/Δ* null mutant had decreased ergosterol production and accumulation of an ergosterol intermediate with similarity to 4-methyl-episterol, also known as gramisterol or 24-methylene-lophenol (**current Fig 6B and C, Sterol A**). The null mutant is the only strain with this ergosterol intermediate, leading us to conclude that this intermediate, in addition to reduced ergosterol, is promoting increased sensitivity to SDS.

Additionally, as we describe in the response above, we performed a more comprehensive and quantitative analysis of the SDS sensitivity using a modified MIC assay, with increasing concentrations of SDS. The homozygous deletion exhibited a 10-fold increase in sensitivity to SDS compared to wild-type control.

Reviewer #3:

1/ Fig 3 and elsewhere, is carrying capacity the same as cell yield? I always thought cell yield was standard.

Carrying capacity here is defined as the maximum population size reached during the 48hr growth curve analysis, measured by optical density (OD_{600}). Whereas cell yield is defined as the number of cells produced per unit of nutrients. We used OD because it allowed us to perform higher throughput growth comparisons between strains and environments, and it allowed us to compare growth phenotypes with antifungal MIC assays, where the standard is to use OD measurements.

2/ Fig 6A, at the far right, I thought 14-methyl ergosta-8-24-dienol was a synonym for 14-methyl-fecosterol. Doesn't erg3 convert 14-methyl fecosterol to the diene diol??

To clarify, 14-methyl ergosta-8-24-dienol is not a synonym for 14-methyl-fecosterol. Instead,14-methyl-fecosterol is the precursor to 14-methyl ergosta-8-24-dienol. Erg3, as the last enzyme of the alternative ergosterol pathway, converts 14-methyl fecosterol into 14-methyl ergosta-8-24-dienol which is the toxic sterol dienol that inhibits fungal cell growth (Kelly et al 1997, Lupetti et al 2002). Unlike the toxic 14-methyl ergosta-8-24-dienol, the accumulation of 14-methyl-fecosterol supports cell growth in presence of fluconazole and this happens when Erg3 is disrupted (Kelly et al.1997, Martel et al. 2010).

3/ I was very impressed by line 230 where the combination of fluconazole and radicicol was fungicidal, and initially I thought it deserved a regular figure, but after looking at Fig S2, I think the verbal description presented is adequate.

Thank you! We appreciate the positive feedback.

Reviewer #4:

(No Response)
